# Advertisement of antibiotics for upper respiratory infections and equity in access to treatment: a cross-sectional study in Nepal

**DOI:** 10.1186/s40545-020-0202-1

**Published:** 2020-02-21

**Authors:** Pramesh Koju, Stéphane P. Rousseau, Marc Van der Putten, Archana Shrestha, Rajeev Shrestha

**Affiliations:** 1 Dhulikhel Hospital, Kathmandu University Hospital, Dhulikhel, PO Box 11008, Kathmandu, Nepal; 2 School of Global Studies, Thammasat University, Bangkok, Thailand; 3 Center of Excellence in Global Health, Faculty of Public Health, Thammasat University, Bangkok, Thailand

**Keywords:** Affordability, Antibiotics, Community pharmacies, Nepal, Promotional activities

## Abstract

**Background:**

Pharmaceutical companies actively advertise their branded antibiotics, which influence their sales at community pharmacies. The major proportion of out of pocket health spending is on medicine; and affordability of antibiotics has always been a crucial issue in most developing countries. This study identified promotional activities adopted by pharmaceutical companies in community pharmacies and medicine shops and the affordability of selected antibiotics to clients with lowest wages in Kavrepalanchok district of Nepal.

**Methods:**

A cross-sectional study was conducted among all community pharmacies and medicine shops (*n* = 34) in Dhulikhel and Banepa. Available pharmacists / personnel were interviewed, using a structured questionnaire, on the characteristics of the pharmacies, promotional activities, and sales and prices of antibiotics used to treat acute upper respiratory tract infections. This study looked at the association of promotional activities (financial bonus, free samples, and books/brochure/gifts) with the type of antibiotics. Further, affordability was assessed of the most popular antibiotics by comparing the total treatment cost against the lowest wage for unskilled workers in Nepal.

**Results:**

Financial bonus, free samples, and brochures were the most popular promotional activities. It is also noticed that antibiotics which are top selling were those with a high number of promotional activities. Amoxicillin, azithromycin and amoxicillin+clavulanate had 42, 29 and 17 promotional activities respectively. Irrespective of the prices of antibiotics, almost all of the most popular antibiotics for acute upper respiratory infections were unaffordable for unskilled workers costing them more than a day’s wage.

**Conclusions:**

Upper respiratory tract antibiotics are widely promoted at community pharmacies. The treatment cost of antibiotics is unaffordable for unskilled workers in Nepal irrespective of the type and unit cost of antibiotics.

## Introduction

Although antibiotics are classified as prescription-only drugs, in Nepal they are widely sold as over-the-counter drugs for upper respiratory tract infections. It is common in Nepal for people with ill health to self-medicate with antibiotics or consult with sales persons at a medicine shop or community pharmacy rather than visiting a licensed health practitioner. There are 51 allopathic pharmaceutical manufacturing companies and 280 foreign pharmaceutical companies in Nepal that distribute the same antibiotics under different brand names [1]. Though these antibiotics have the same composition, the prices vary by brand. Pharmaceutical companies provide financial incentives to community pharmacies and local medicine shops to boost sales of expensive antibiotics [2]. The pharmaceutical companies compete for a larger market share through extensive advertisements, gifts, free medicine samples, and financial bonuses to doctors and community pharmacies [3]. The cost of advertising is included in the selling price, as part of the cost of production, distribution and marketing, and so forms part of the price paid by the consumer [4]. A study conducted in 2009 in Nepal reported that expensive antibiotics were prescribed and dispensed more often than the cheaper ones [5]. In 2002 pharmaceutical companies spend at least 30 times more on medicine promotion than on medicine information [6].

The overuse of antibiotics causes antimicrobial resistance and inequitable access to health care [7, 8]. One-third of the global population lack access to medicines [9], with up to 50% of the affected population in Asia and Africa [10]. People spend up to 70% of overall health spending on medicine in low and middle income countries (LMIC), compared to 10–18% in high income countries (HIC) [11]. Most of the people in LMIC like Nepal pay out-of-pocket for these medicines. However, one-third of the LMIC either have no regulatory authority or limited capacity to regulate the medicine market [12]. The price of the same medicine manufactured by different pharmaceutical companies varies. Whereas prices of same medicines varies between countries as well [13]. Considering the landscape of pharmaceutical companies in Nepal and the country’s relative small market, there is competition between domestic companies and international companies to sell their brands. Despite the benefits of the promotional activities, sales might be affected by the community pharmacies’ trust in specific brands based on their experiences [14]. Nepal’s Department of Drug Administration (DDA) has developed a Guideline on Ethical Promotion of Medicine (2007) to enhance ethical practice in the delivery of healthcare. Unfortunately implementation of the guideline faces challenges due to conflict of interest among various stakeholders [4]. Moreover, consumer law and protection agencies in LMIC are less effective compared to HIC. This study identified different forms of advertising activities adopted by pharmaceutical companies in community pharmacies and assessed the affordability of those antibiotics among customers earning the lowest wages in Kavrepalanchok district of Nepal.

## Methods

A cross-sectional study was conducted among all community pharmacies (*n* = 34) listed by the DDA of Nepal in two towns of Kavrepalanchok district of Nepal. The district and the towns were purposively selected using following criteria: the investigator was acquainted with the district and had a well-established network with local stakeholders including community pharmacies; these areas were relatively more developed than other areas of the district; and they had an infrastructure that included educational institutes, hospitals, health centers, and community pharmacies. Interview respondents were those present in the community pharmacies at the time of the survey (August 15 to August 29, 2016). The survey used a structured questionnaire. The Ethical Review Committee of Thammasat University, Thailand approved the study. Confidentiality was maintained by replacing personal identifiers with a respondent code and using a password protected electronic database.

Experts evaluated the draft questionnaire for face validity and the questionnaire was pretested before the survey using a test-retest reliability technique. Data were collected on different forms of promotional activities (i.e. brochures/books/gifts, financial bonus, free samples) for the top three selling branded and generic antibiotics for acute upper respiratory tract infection (amoxicillin, amoxicillin+clavulanate and azithromycin) from all community pharmacies in two towns of Kavrepalanchok district.

Characteristics of the participants were described using frequencies and proportions. Further, the association of types of antibiotics with promotional activities were determined using descriptive analysis. Data were analyzed using a case function in SPSS.

Affordability was determined by comparing the total cost of standard treatment against the lowest wage for unskilled workers working in all enterprises excluding those working in tea farms and the jute industry in Nepal at the time of the survey based on rates published in the Nepal Gazette, 2009 [15]. Treatment costing 1 day wage or less for a standard treatment (full course of treatment) for an acute condition was considered affordable [10]. Data was analyzed using (SPSS) version 21.0.

## Results

The characteristics of the study respondents are presented in Table 1. All the pharmacies were privately owned. Thirty-five percent did not have education background in pharmaceutical science (whether a diploma, bachelor or master degree in pharmacy science). More than half of the respondents (56%) had been practicing pharmacy for less than 5 years. Most of the respondents (76%) were the owners themselves.

**Table 1:** Demographic Profile of Respondents

	Number	Percentage (%)
Pharmacy Setting		
Private Pharmacy	34	100
Education		
Health Assistant Degree	11	32.4
Community Medical Auxiliary	1	2.9
Diploma in Pharmacy	9	26.5
Bachelor’s in Pharmacy	12	35.3
Master’s in Pharmacy	1	2.9
Number of years in working in pharmacy		
Less than 2 years	8	23.5
2–5 years	11	32.4
5–10 years	6	17.6
More than 10 years	9	26.5
Relationship with owner		
I am the owner	26	76.5
A relative	2	5.9
An employee	6	17.6

**Fig. 1:**
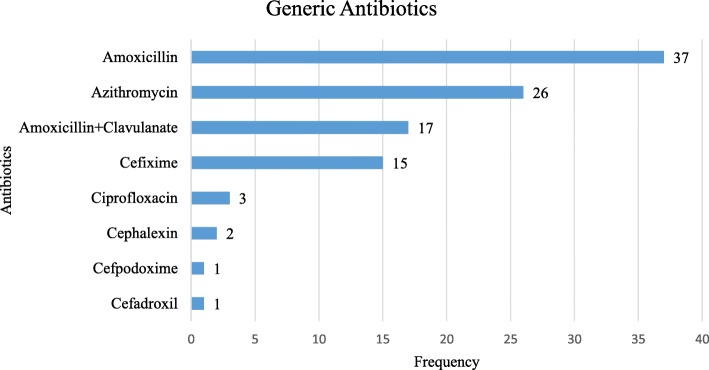
Frequency of Generic Antibiotics Sold in Community Pharmacies

Eight types of generic antibiotics were sold in the community pharmacies (Fig. 1). Amoxicillin was the most sold generic antibiotic with the highest count (37 times), followed by azithromycin (26 times) and amoxicillin+clavulanate (17 times).

Looking at promotional activities for the generic antibiotics (Table 2), it was clear that the antibiotics which were top selling were those with a high number of promotional activities. In total, amoxicillin, azithromycin, amoxicillin+clavulanate had 42, 29 and 17 promotional activities respectively. The highest number of promotional activities was observed for amoxicillin followed by azithromycin and amoxicillin+clavulanate with a substantial number of financial bonus, free samples, and brochures. Promotion through financial bonus was most popular among all the generic antibiotics except for cefpodoxime, whereas promotions in kind (e.g. offering books) were only seen in one antibiotic, being the least popular means of promotion.

**Table 2:** Frequencies of Forms of Promotional Activities per Generics

S. No	Generic Name	Promotional Activities					Total
		Brochures	Books	Gifts	Financial Bonus	Free Sample	
1	amoxicillin	7	0	4	16	15	42
2	azithromycin	12	0	2	7	8	29
3	amoxicillin+clavulanate	2	0	1	6	8	17
4	cefixime	4	1	0	4	6	15
5	ciprofloxacin	0	0	0	1	2	3
6	cephalexin	0	0	0	1	0	1
7	cefadroxil	0	0	0	1	0	1
8	cefpodoxime	0	0	0	0	1	1
	Total	25	1	7	36	40	109

The price among different brands ranged from 10 to 55 Nepalese Rupees (1 United States Dollar, USD = 110 Nepalese Rupees, NPR) (Table 3). For the treatment of streptococcal pharyngitis, two antibiotics were not affordable [amoxicillin required 1.26 days’ wage and amoxicillin+clavulanate required 4.7 days’ wage]. For the treatment of acute bacterial sinusitis, two of the selected antibiotics were unaffordable i.e. amoxicillin required 1.35 days’ wages followed by 9.8 days’ wages for amoxicillin+clavulanate. In the case of pertussis, azithromycin costed 1.4 days’ wage, which was also unaffordable.

**Table 3:** No. of Day’s Wages of Unskilled Worker Needed to Purchase Standard Treatments

Indications	Antibiotics	Strength (Tablet/Capsule)	No. of units a day	Duration days	Mean Price (Rs)	No. of units a day × Duration of days × Mean price (Rs)	Day’s wages to pay for treatment
Streptococcal pharyngitis	amoxicillin	500 mg	2	10	10	200.0	1.26
	amoxicillin + clavulanate	500 to 875 mg	2	10	35.9	717.6	4.7
	azithromycin	500 mg	1	3	42.31	126.9	0.8
Acute bacterial sinusitis	amoxicillin	500 mg	3	5–7	10	210.0	1.35
	amoxicillin + clavulanate	625 mg	3	14	35.88	1506.9	9.8
	azithromycin	500 mg	1	3	42.31	126.93	0.83
Pertussis	azithromycin	500 mg	1	5	42.31	211.5	1.4

## Discussion

To the authors’ knowledge, this study is first in Kavrepalanchok, Nepal to look at promotional activities in sales of antibiotics and the affordability of antibiotics for unskilled workers. Eight types of generic antibiotics were sold in community pharmacies for the treatment of acute upper respiratory tract infection. The top three sales for antibiotics were amoxicillin, azithromycin, and amoxicillin+clavulanate. The promotional activities for the top selling antibiotics were found to have high number of promotional activities. All of the highest selling antibiotics for the treatment of streptococcal pharyngitis, acute bacterial sinusitis and pertussis were unaffordable for unskilled workers; except azithromycin for streptococcal pharyngitis and acute bacterial sinusitis.

Survey findings showed that 65% of the respondents had an educational background in pharmaceutical science and that 32.4% held a health assistant degree which illustrates progress in the provision of a qualified health workforce compared to findings of previous studies in Nepal [16, 17]. Community pharmacies increasingly employ people with a background in pharmaceutical science and pharmacy staff better understands the practice of proper dispensing and counseling. All of the pharmacies are private owned similar to other studies in Nepal [18, 19]. In addition, findings in this illustrated that the majority of community pharmacies’ respondents were owners, which might be due to increasing interest in and scope for self-employment among pharmacists fueled by the lack of employment opportunities as hospital-based pharmacist, industrial pharmacist, teaching positions etc.

In this survey, eight types of common antibiotics were observed. Among these eight antibiotics, six were listed in the WHO model list of essential medicines [20], whereas two, cefpodoxime and cefadroxil, were not found in the WHO list, however these were recorded only once in this survey. Similar in the study, amoxicillin was observed to be most commonly used over the counter antibiotic [21]. It could be due to relatively low cost and broad spectrum activity [22], where the cost is observed to be low in the study as well. Further, the three top-selling generic antibiotics identified by the survey were listed in the National List of Essential Medicine (NLEM), Nepal, which comprises the essential medicines for the treatment of most prevailing diseases in the country [23]. In the NLEM, the top two selling antibiotics, amoxicillin and azithromycin, were recorded in the main list whereas the amoxicillin+clavulanate combination was listed in the complementary list. It should be noted that these antibiotics were used mostly for indications of upper respiratory infections. According to the guidelines for the use of antibiotics in acute upper respiratory tract infections, the three selected antibiotics are used either as a first line therapy or alternative therapy [24].

This study reported several promotional activities in the areas, such as offering free samples, financial bonuses, and brochures. The use of free samples and financial bonuses was widely observed in this study to promote various antibiotics although the national drug regulatory authority of Nepal discourages unethical promotional practices including financial bonus. Although the Government of Nepal, Department of Drug Administration (DDA), released Guidelines on the Ethical Promotion of Medicine (2007), implementation faced challenges due to conflict of interests among stakeholders [25]. Whereas regulations on advertisement and promotional materials for medicines is enforced in many countries in the world [26], LMIC like Nepal face challenges in implementing such regulations. One third of the LMIC have no regulatory authority or inadequate capacity to control the drug market [12]. A systematic review done in LMIC countries stated that there is an aggressive marketing involving promotional activities to pharmacies by the pharmaceutical companies [27]. A study conducted in India also showed that retailers are incentivized with bonus schemes for selling their products by pharmaceutical companies [28]. An earlier study conducted in Nepal, established that pharmaceutical companies try to incentivize community pharmacies by offering gifts and bonuses through medical representatives (MR) [5]. Moreover, there is no law in Nepal that regulates the content of promotional materials provided by pharmaceutical companies [29]. Provision of financial bonus was found to be most popular in this study which has also been observed in other studies [2, 5, 30]. Whereas some studies reported that financial grants and valuable gifts were provided to doctors and retailers for dispensing prescription drugs [31]. In addition to that, in LMIC small pharmacies were found to be dispensing more expensive over the counter antibiotics to make profit [21]. One of the studies conducted in Nepal reported that retailers / community pharmacies were offered bonuses if they promoted or substituted medicine brands [5]. The promotional activities might also been flourished due to highly competitive market which depends in quality, pricing and advertisement. In addition to the promotion activities, antibiotic being the more profitable medicines, community pharmacies are highly encouraged to sell the antibiotics [32].

In this study, all antibiotics were unaffordable to unskilled workers, except azithromycin for the treatment of streptococcal pharyngitis and acute bacterial sinusitis. While the analysis included only the price of medicine, treatment would be even more unaffordable if the costs for doctor’s honorarium and diagnostic tests were included. The affordability of medicine might also depend upon the availability of cheaper alternatives. Given that 25.2% of Nepal’s population is living below the national poverty line, defined as a person per-capita total annual consumption below 19,261 Nepalese Rupees [33] and 24.5% of the population living below international poverty line, defined as an income of 1.25 USD a day [34], the treatments are far more costly for a substantial proportion of the population. In addition, those living with chronic diseases, needing lifelong treatment, requiring multiple therapies of antibiotics and those families who have more than one of its members needing these treatments face an additional burden [35]. Studies showed that families often have to rely on taking a loan at high interest rates from local money lenders or selling property [11]. Whereas a systematic review conducted on LMIC Asian settings revealed that patients requested and buy incomplete courses of antibiotics due to economic constraints [27].

Pharmaceutical companies spend a large amount of their resources on marketing and advertisement [36]. The money spent by companies on these marketing and promotional activities will be included in the medicine price, as part of the production and distribution cost, that is ultimately paid by the consumers [5]. The consumers with low-income are constrained in accessing the recommended dose regimen and often end up buying a proportion of the required regimen of antibiotics eventually resulting in an irrational use of drugs. In such instances, the irrational use of antibiotics leads to antibiotic resistance and eventually resorting to much more expensive 2nd and 3rd line treatments including hospitalization, which will hike the expenses for patients, affecting affordability among the poor. The World Health Organization (WHO) reported that 80% of antibiotics are used in community where 20–50% are used inappropriately in LMICs [37]. Hence, the impact of promotional activities may have severe implications on the irrational use of drugs and therefore ultimately adversely affect health equity among the vulnerable. Further studies on the impact of promotional activities on irrational use of drugs and its consequences on health costs should be conducted. A systematic review conducted on WHO Southeast Asian region also noted that there is a high need of well-designed study on use of antibiotics in community for the effective planning and design implementing strategies / interventions to prevent irrational antibiotic use and constraint the antibiotic resistance [38], which is causing a major public health implications including prolonged hospital stays, long term disability, significant additional costs to health systems, high costs for patients and families.

### Limitations

It is acknowledged that this study has some limitations. For example, the study did not include a detailed analysis of promotional activities across brands of antibiotics. Secondly, promotional activities might have been underreported by community pharmacies. Further, the study was conducted in two cities of Kavrepalanchok district, and therefore cannot be generalized to Nepal. Finally, the study did not assess the impact of promotional activities on the sales of specific antibiotics. However, the study findings discussed below provide a benchmark for future research highly relevant to healthcare practice in LMIC.

## Conclusion

The use of antibiotics for upper respiratory tract infections is promoted using financial bonus, free samples, and brochures as the most popular means of promotions. Irrespective of the unit price of antibiotics, almost all of the most popular antibiotics were unaffordable for unskilled workers costing them more than a day’s wage. The concerned national drug regulatory authority of the government of Nepal and other government stakeholders should monitor and control the promotional activities and drug prices to ensure affordability and accessibility for the poor segment of the population. Further study is recommended on promotional activities and their effect on the sales and price of antibiotics involving customers, community pharmacies, marketing representatives, executives of pharmaceutical industries, and authorized personnel from the government sector.

## Data Availability

Data are available from the corresponding author upon reasonable request.

## Abbreviations

DDA: Department of Drug Administration
HIC: High Income Countries
LMIC: Low and Middle Income Countries
NLEM: National List of Essential Medicine
NPR: Nepalese Rupees
SPSS: Statistical Package for the Social Science
USD: United State Dollar
